# Comparison of different pain scoring systems in critically ill patients in a general ICU

**DOI:** 10.1186/cc6789

**Published:** 2008-02-16

**Authors:** Sabine JGM Ahlers, Laura van Gulik, Aletta M van der Veen, Hendricus PA van Dongen, Peter Bruins, Svetlana V Belitser, Anthonius de Boer, Dick Tibboel, Catherijne AJ Knibbe

**Affiliations:** 1 Department of Anaesthesiology and Intensive Care, St. Antonius Hospital, Koekoekslaan 1, Nieuwegein, 3440 EM, The Netherlands; 2 Department of Clinical Pharmacy, St. Antonius Hospital, Koekoekslaan 1, Nieuwegein, 3440 EM, The Netherlands; 3 Department of Pediatric Surgery, Erasmus Medical Centre, Sophia Children's Hospital, Dr. Molewaterplein 60, Rotterdam, 3015 GJ, The Netherlands; 4 Department of Pharmacoepidemiology and Pharmacotherapy, Faculty of Pharmaceutical Sciences, University of Utrecht, Sorbonnelaan 16, Utrecht, 3584 CA, The Netherlands

## Abstract

**Background:**

Pain in critically ill patients in the intensive care unit (ICU) is common. However, pain assessment in critically ill patients often is complicated because these patients are unable to communicate effectively. Therefore, we designed a study (a) to determine the inter-rater reliability of the Numerical Rating Scale (NRS) and the Behavioral Pain Scale (BPS), (b) to compare pain scores of different observers and the patient, and (c) to compare NRS, BPS, and the Visual Analog Scale (VAS) for measuring pain in patients in the ICU.

**Methods:**

We performed a prospective observational study in 113 non-paralyzed critically ill patients. The attending nurses, two researchers, and the patient (when possible) obtained 371 independent observation series of NRS, BPS, and VAS. Data analyses were performed on the sample size of patients (*n *= 113).

**Results:**

Inter-rater reliability of the NRS and BPS proved to be adequate (kappa = 0.71 and 0.67, respectively). The level of agreement within one scale point between NRS rated by the patient and NRS scored by attending nurses was 73%. However, high patient scores (NRS ≥4) were underestimated by nurses (patients 33% versus nurses 18%). In responsive patients, a high correlation between NRS and VAS was found (r_s _= 0.84, *P *< 0.001). In ventilated patients, a moderate positive correlation was found between the NRS and the BPS (r_s _= 0.55, *P *< 0.001). However, whereas 6% of the observations were NRS of greater than or equal to 4, BPS scores were all very low (median 3.0, range 3.0 to 5.0).

**Conclusion:**

The different scales show a high reliability, but observer-based evaluation often underestimates the pain, particularly in the case of high NRS values (≥4) rated by the patient. Therefore, whenever this is possible, ICU patients should rate their pain. In unresponsive patients, primarily the attending nurse involved in daily care should score the patient's pain. In ventilated patients, the BPS should be used only in conjunction with the NRS nurse to measure pain levels in the absence of painful stimuli.

## Introduction

Pain is a frequently experienced problem in critically ill patients in the intensive care unit (ICU) [1]. Pain may increase morbidity and mortality and may decrease the comfort of patients and health-related quality of life. The adequate use of analgesics and sedatives therefore may decrease morbidity and mortality [2]. Measurement of pain in ICU patients, however, may be complicated by decreased consciousness, severity of illness, mechanical ventilation, and the use of sedatives in these patients, particularly when high doses of sedatives are administered [3,4]. Although self-report is still the 'gold standard' in pain measurement according to the guidelines of the International Association for the Study of Pain [5], one segment of ICU patients is unable to communicate effectively. In these cases, the gold standard (that is, the pain intensity reported by the patient) is not possible or is potentially unreliable. This is also a common problem in, for example, neonates and children, who are not able to report pain in a reliable manner [6].

Therefore, pain assessment in the ICU remains a challenge for clinicians and researchers. There is no specific neurobiological parameter for the evaluation of pain, nor does an objective quantification of pain intensity or relief exist [7]. Various pain scales are available, but it remains unclear whether they can be applied reliably in the diverse patient population of the ICU, where patients not only may be mechanically ventilated but also are subject to repeated painful procedures. Therefore, it is of interest to define which score should be used for which patient (for example, ventilated, responsive, or unresponsive) and by which health care worker, in case the patient cannot communicate. These results can be used to implement a systematic evaluation of pain in all ICU patients. While, to date, the use of scoring systems for pain severity and sedation depth and the implementation of protocols increase with a more patient-oriented regime for analgesia and sedation, a trend is observed away from a hypnosis-based approach and toward an analgesia-based approach. Although these changes may improve pain and sedation practice, further efforts are needed for widespread implementation of pain scoring systems and analgesia protocols [8,9]. Of the available pain scales, the Numerical Rating Scale (NRS) (1 to 10) and the Visual Analog Scale (VAS) (1 to 100) have been validated for acute pain only and not in mechanically ventilated patients in the ICU [10]. The Behavioral Pain Scale (BPS) was developed specifically for measuring the severity of pain in sedated, mechanically ventilated, unresponsive patients [11], but this pain scale still is not generally accepted for routine use. Another question in pain management in the ICU is which health care worker (nurse, physician, and so on) should rate pain in case the patient cannot communicate verbally. While the attending nurse is involved in close daily care of the patient, the physician seems to have a more distant relation to the patient. Therefore, we designed a study (a) to determine the inter-rater reliability of the NRS and BPS, (2) to compare pain scores of different observers and the patient, and (c) to compare NRS, VAS, and BPS for measuring pain in ventilated and non-ventilated patients in the ICU.

## Materials and methods

### Design

A prospective observational study was performed in a 30-bed surgical/medical ICU in a teaching hospital in Nieuwegein, The Netherlands. The medical ethical committee of St. Antonius Hospital approved the study protocol and waived the need for informed consent because the observational study design and pain measurement are part of the standard care.

### Participants

All patients in the ICU who were at least 18 years old were included between 27 June and 4 August 2005. Patients who received neuromuscular blocking medications or muscle-paralyzing drugs continuously, who were unconscious after resuscitation, who were quadriplegic, who suffered from a critical illness (poly)neuropathy, or who had an epidural catheter were excluded. Paralysis, whether caused by a pre-existing condition or by medication, makes the BPS unreliable.

### Pain measurement instruments

To assess pain intensity, three pain scales (that is, BPS, NRS, and VAS) were used. The BPS is used after an observation of the patient for about 1 minute and was validated in critically ill, sedated, and mechanically ventilated patients [10,11]. The BPS is a pain scale for sedated and ventilated patients exclusively and is based on the sum of three subscales: facial expression, upper limb movements, and compliance with mechanical ventilation (Table 1). Each subscale is scored from 1 (no response) to 4 (full response). Therefore, BPS scores range from 3 (no pain) to 12 (maximal pain) [10,11]. The BPS has a maximal acceptable pain score of 5 [12]. The NRS is based on a scale from 0 to 10; 0 represents no pain and 10 represents the worst possible pain [13,14]. The NRS has a maximal acceptable pain score of 3 [15]. The VAS is a 100-mm ruler with a movable cursor. At the left side is written 'no pain' and at the right side is written 'worst possible pain'. The patient marks the intensity of pain [16,17]. The VAS has a maximal acceptable pain score of 30 mm.

**Table 1:** The Behavioral Pain Scale [15]

Item	Description	Score
Facial expression	Relaxed	1
	Partially tightened	2
	Fully tightened	3
	Grimacing	4
Upper limbs	No movement	1
	Partially bent	2
	Fully bent with finger flexion	3
	Permanently retracted	4
Compliance with ventilation	Tolerating movement	1
	Coughing but tolerating ventilation for most of the time	2
	Fighting ventilator	3
	Unable to control ventilation	4

### Depth of sedation

The Ramsay Scale (RS) was used to assess the sedation level [18]. The RS is a scale from 1 to 6, with higher levels indicating increased degrees of sedation, and considers the following levels: (1) patient anxious, agitated, and restless; (2) patient cooperative, orientated, and tranquil; (3) patient drowsy or asleep and responds easily to commands; (4) patient asleep and gives a brisk response to a light glabellar tap; (5) patient asleep and gives a sluggish response to a light glabellar tap; and (6) patient asleep and gives no response to a light glabellar tap [19].

### Standard pain medication in the intensive care unit

All patients received pain medication according to the local standard protocol, consisting of 1 gram of acetaminophen rectally three times daily and 10 mg of morphine subcutaneously four times daily or 30 to 50 mg of morphine per day using a continuous intravenous infusion, if required.

### Procedures

Before this study, levels of pain were not systematically scored and recorded. During this study, assessments took place in all patients in the ICU twice a day (at 8.30 a.m. and 3 p.m.) during 1 month. Assessments were initiated by two researchers who were trainees in pharmacy and who had been working for 6 months under close supervision of two anesthesiologists of the Department of Anaesthesiology/Intensive Care and one ICU nurse. These researchers were not involved in the patient's care but took notice of the clinical and medical situation of the patient, similar to a physician on ward rounds. All assessments were made during non-nociceptive procedures in order to obtain basal pain scores. First, the researchers observed every patient for about 1 minute. Assessments of the researchers were made simultaneously but were independent of each other. Then, the researchers scored the BPS, NRS, and RS in order to prevent the outcome from being influenced by the patient's or nurse's score. The BPS was scored only in patients who were ventilated. Then, the attending nurse was asked to score the pain of the patient with the NRS. If the patient was responsive, the patient was asked to score the pain using the NRS and VAS. Gender, height, weight, year of birth, ICU indication, and relevant history were collected. Patients were classified in one of the two ICU indications, 'cardiothoracic surgery' or 'non-cardiothoracic surgery, with a skewed distribution for 'cardiothoracic surgery'. In this study, the 'NRS researcher' and 'BPS researcher' are defined by NRS rating and BPS rating by the researcher. The 'RS researcher' is defined by the RS rating by the researcher. 'NRS nurse' is defined by the NRS rating by the nurse. 'NRS patient' and 'VAS patient' are defined by the NRS rating and VAS ratings by the patient (Table 2).

**Table 2:** Pain and sedation scales performed by researcher, nurse, and patient

	Behavioral Pain Scale (if ventilated)	Numerical Rating Scale	Visual Analog Scale	Ramsay Scale
Researcher	×	×		×
Nurse		×		
Patient (whenever possible)		×	×	

### Training pain measurement instruments

For adequate use of the BPS, NRS, VAS, and RS, the two researchers attended a 4-hour training session (conducted by a trained ICU nurse), during which the BPS and RS were explained with examples of patients who were in the ICU at the time of the training. When the inter-rater reliability was acceptable according to a quadratic weighted Cohen's kappa of greater than 0.6, the researchers were allowed to score patients for the study [20].

### Data analysis

Data were analyzed with the statistical software S-Plus^® ^version 6.2 (Insightful Corporation, Seattle, WA, USA). To correct for the different numbers of measurements per patient, one observation per patient was randomly selected. All statistical analyses were performed using this independent sample, while all measurements were plotted in the figures for better illustration. Kappa coefficients with quadratic weights were used to reflect agreement for ordinal scales (NRS, BPS, and RS) between the independent researchers. Weighted kappa penalizes disagreement in terms of their seriousness [20]. Theoretically, the value of kappa can range from 0 (disagreement) to 1 (perfect agreement). A value larger than 0.6 was regarded as satisfactory [21]. The 95% confidence intervals for kappa coefficients were calculated. Spearman non-parametric rank correlation coefficients (r_s_) were used to measure the degree of correlation for two ordinal variables. The null hypothesis that the correlation coefficient is zero was tested. A *P *value of less than 0.05 was considered statistically significant.

## Results

### Patient characteristics and data

A total of 138 intensive care patients entered the study, with a median of two observation series per patient (range 1 to 15). In total, 25 patients were excluded (15 patients because of incomplete collection of the data and 10 patients because of exclusion criteria), resulting in a total of 113 included patients. Table 3 shows the baseline characteristics of the patients. The body mass index was recorded for 87 of 113 patients (77%). In total, 371 observations were scored by the researchers and 322 observations were scored by the nurses. In a total of 75 patients (180 observations), the patient could report his or her pain using the NRS. In 141 observations, the patient could also report his or her pain using the VAS. Of the 57 ventilated patients, 13 patients were communicative and could report their pain.

**Table 3:** Baseline patient characteristics

Number of patients	113
Age, years	66 ± 15^a^
Male gender, number	78 (69%)
Body mass index, kg/m^2^	26.6 ± 4.7^a^
Diagnostic categories, number	
Cardiothoracic surgery	83 (73%)
Non-cardiothoracic surgery	30 (27%)
Mechanical ventilation, number	
None	56 (50%)
Pressure-support	36 (32%)
Volume-controlled	21 (19%)
Median Ramsay Scale score (range)	2.0 (1.0–5.0)

^a^Values are expressed as mean ± standard deviation.

#### Inter-rater reliability

Table 4 depicts the exact agreement, the agreement within one scale point, and the quadratic weighted kappa for the NRS, BPS, and RS in different groups of patients for the two independent researchers. There was no difference between the ICU indications 'cardiothoracic surgery' (*n *= 83) and 'non-cardiothoracic surgery' (*n *= 30) in exact agreement (60% versus 57%) and agreement within one scale point (94% versus 93%).

**Table 4:** Inter-rater reliability of the NRS, BPS, and Ramsay Scale

Agreement between	Exact agreement	Agreement within 1 scale point	Kappa (CI)	Number
All patients				
NRS	59%	94%	0.71 (0.61–0.81)	113
BPS	80%	100%	0.67 (0.54–0.80)	57
Ramsay Scale	91%	100%	0.66 (0.36–0.97)	44
Non-ventilated patients				
NRS	66%	95%	0.63 (0.45–0.82)	56
Volume-controlled ventilated patients				
NRS	56%	94%	0.64 (0.48–0.81)	36
BPS	81%	100%	0.59 (0.36–0.82)	36
Pressure-support ventilated patients				
NRS	48%	90%	0.80 (0.68–0.93)	21
BPS	81%	100%	0.76 (0.62–0.90)	21
Cardiothoracic patients				
NRS	60%	94%	0.65 (0.53–0.77)	83
Non-cardiothoracic patients				
NRS	57%	93%	0.80 (0.65–0.93)	30

BPS, Behavioral Pain Scale; CI, confidence interval; NRS, Numerical Rating Scale.

#### NRS patient versus NRS nurse or NRS researcher

**Figure 1:**
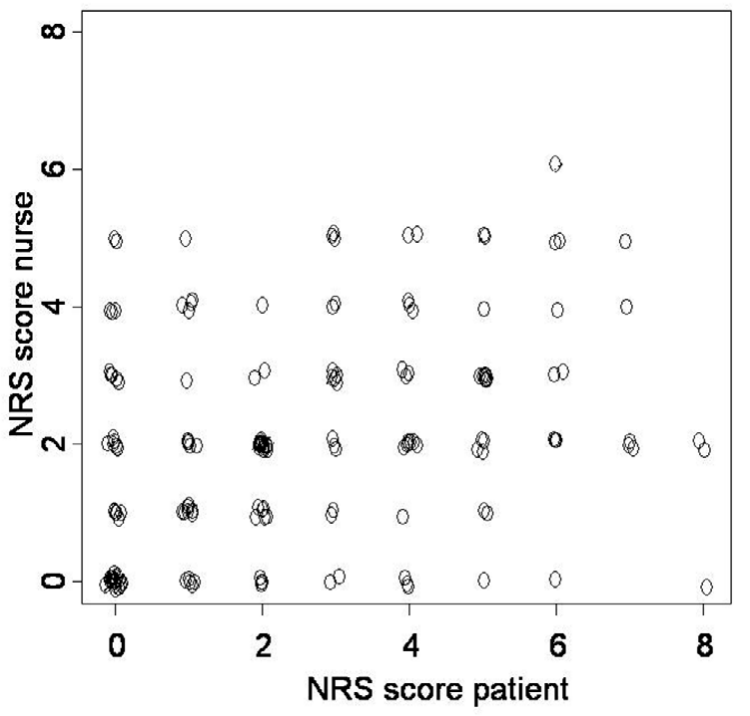
Correlation between Numerical Rating Scale (NRS) scores of patient and nurses. Data of 75 responsive patients with 165 measurements are presented (with x = y line).

For the patients who were able to report their own pain levels (*n *= 75), the level of agreement within one scale point between NRS patient and NRS nurse was 73% compared with 58% for the NRS researcher, corrected for multiple observations per patient. Similar results were found for the exact agreement (42% versus 19%, respectively). The correlations between NRS of patient and nurses (Figure 1) and between NRS of patient and researcher were moderate and low, respectively (r_s _= 0.55, *P *< 0.001 versus r_s _= 0.38, *P *= 0.009). Whereas 33% of the patients scored NRS values of greater than or equal to 4, only 18% of the attending nurses scored NRS values in that range. Apparently, particularly when the patient rated his or her pain as unacceptable, nurses tended to underestimate the pain level of the patient on the NRS.

#### NRS patient and VAS patient

**Figure 2:**
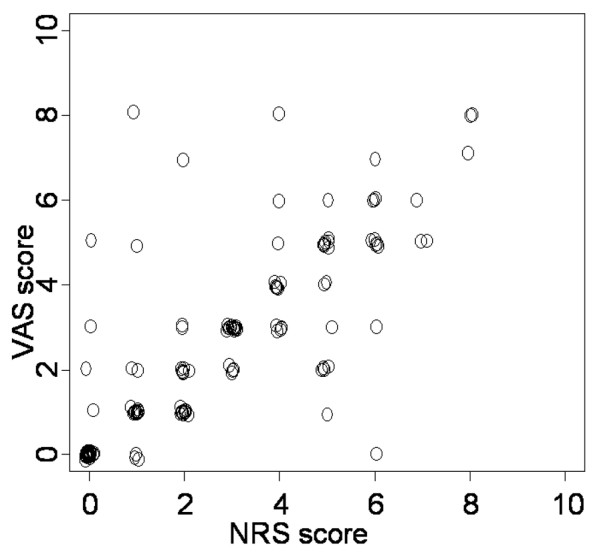
Correlation between Numerical Rating Scale (NRS) score and Visual Analog Scale (VAS) score of the patient. Data of 75 responsive patients with 131 measurements are presented.

In responsive patients, there was a strong positive correlation between the NRS patient and the VAS patient (r_s _= 0.84, *P *< 0.001, *n *= 75; Figure 2). The correlation between NRS patient and VAS patient was slightly lower in cardiothoracic patients than in non-cardiothoracic patients (r_s _= 0.79, *P *< 0.001, *n *= 25 patients versus r_s _= 0.95, *P *< 0.001, *n *= 11 patients).

#### NRS nurse and BPS researcher

**Figure 3:**
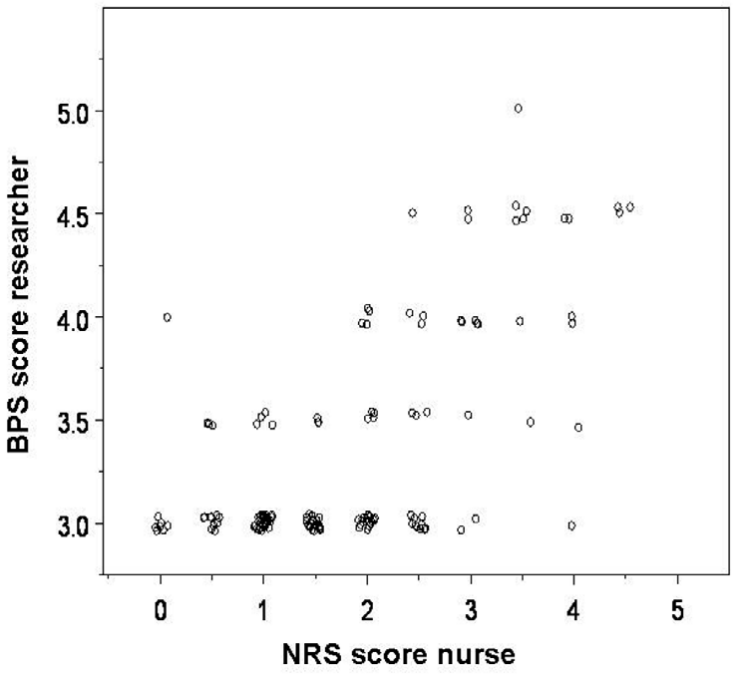
Correlation between Numerical Rating Scale (NRS) score and Behavioral Pain Scale (BPS) score. Data of 57 ventilated patients with 151 measurements are presented.

While a moderate positive correlation was found between the NRS nurse and the BPS researcher in ventilated patients (r_s _= 0.55, *P *< 0.001, *n *= 57 patients; Figure 3), Figure 3 also shows that using the NRS, only 5% of the observations (*n *= 151 observations of 57 patients) were NRS of 0 (no pain), whereas on the BPS, 68% of the observations were BPS of 3 (no pain). Besides, using the NRS, 6% of the observations were NRS of greater than or equal to 4, considered to be unacceptable pain. However, corresponding BPS scores were all low (median 3.0, range 3.0 to 5.0) and below the acceptable BPS of 5, which means that no unacceptable pain was observed using the BPS. There was no difference in the correlation between cardiothoracic patients and non-cardiothoracic patients (r_s _= 0.54, *P *< 0.001, *n *= 47 patients versus r_s _= 0.53, *P *= 0.047, *n *= 10 patients) or between pressure-supported ventilated patients compared with volume-controlled ventilated patients (r_s _= 0.64, *P *= 0.004, *n *= 21 patients versus r_s _= 0.49, *P *= 0.004, *n *= 36 patients).

#### NRS researcher and Ramsay Scale researcher

The correlation between the NRS researcher and the RS researcher was low (r_s _= 0.28, *P *= 0.078, *n *= 40). The correlation was weak in both cardiothoracic patients and non-cardiothoracic patients (r_s _= 0.24, *P *= 191, *n *= 31 patients versus r_s _= 0.04, *P *= 0.9, *n *= 13 patients).

## Discussion

In our study, we found that the inter-rater reliability for the NRS and BPS was good, which proves that it is possible to train medical personnel to use these scales in a reliable way in ICU patients. However, although the different pain scales show a high reliability, an important finding of our study is that especially unacceptably high patient scores (NRS ≥4) were underestimated by both the nurses and the researchers as 33% of the NRS patient values were equal to or greater than 4 compared with 18% for the nurses. As it is known that the patients may underestimate pain by themselves (caused by factors like culture and the environment [22,23]), the risk of underestimation seems to be an important issue when scoring pain in ICU patients. The underestimation of patients' pain scores by the nurses is already supported in the literature. However, the finding that underestimation occurs in especially high patient scores was never reported. Therefore, it is of utmost importance to use restrictive sedation protocols aiming at cooperative sedation levels instead of unconscious levels [4], allowing for response to questions about pain evaluation and thereby reducing observed-based pain evaluations and allowing for self-report of pain.

The correlation between the NRS and the BPS in our study is in accordance with the study of Payen and colleagues [11], which showed that the BPS is reliable for measuring the severity of pain in sedated and ventilated patients. Also, Aissaoui and colleagues [10] concluded that the BPS is valid and reliable for measuring intervention pain in non-communicative ICU patients. In our study, however, in 57 ventilated patients of which 13 were communicative, only 5% of the observations (*n *= 151 observations of 57 patients) were NRS of 0 (no pain), whereas on the BPS, a remarkable 68% of the observations were BPS of 3 (no pain). In addition, although 6% of our observations were NRS of greater than or equal to 4 (unacceptable pain according to [24]), the BPS scores were all low with a median value of 3.0 (range 3.0 to 5.0), which is the lowest possible value of the BPS in a scale with a maximum value of 12. Also, in the study of Payen and colleagues [11], a high non-response on the BPS was found in assessments completed at rest (BPS score of 3 in 88% to 97% of the observations) and 82% of the observations at rest and during interventions were clustered around BPS scores of 3 to 6.

The high non-response on the BPS can be explained by the short time of observation. During 1 minute of observation, the patient may seem pain-free (BPS of 3). However, using the NRS, a higher score may be rated as the nurse tends to include more background information of the patient (for example, the pain levels from the last hours while caring for the patient). So the BPS reflects the objective visible behavior at one specific time point, whereas the NRS represents a global impression of pain, including several contextual factors during a longer time period. It seems, therefore, that the BPS should be used only in conjunction with the NRS nurse to measure pain levels the ICU.

Various studies concluded that, compared with the NRS, the VAS is not an adequate tool in patients with decreased consciousness [25,26]. This appears to be related to the lack of ability for abstraction and comprehension and provides difficulties in patients who are injured to the upper limbs. In our study, the correlation between the NRS and VAS estimated by the patient is strong (r_s _= 0.84, *P *< 0.001, *n *= 75), suggesting that the VAS is also an adequate tool for measuring pain in about two thirds of the patients in our ICU. However, the VAS could be used in only 75 of 113 patients, in particular in patients with intact comprehension and abstraction, when recovering from critical illness, and just before leaving the ICU following cardiac surgery. Therefore, it is unknown whether this finding can be extrapolated to other ICUs.

The correlation between the NRS score and RS score was low (r_s _= 0.28, *P *= 0.078, *n *= 40). So the degree of pain intensity does not seem to depend on the level of sedation. This is important because these two scores should be able to distinguish between the level of analgesia and the level of sedation. Whereas high RS levels (deep sedation) may be expected in more severely ill patients experiencing more pain, patients with low RS levels (light sedation), in contrast, have more ability to show painful behaviors, resulting in the absence of a significant correlation.

This study had several limitations. In the present study, we collected pain scores of ICU patients at rest and without painful stimuli. In the ideal study design in which different pain scoring systems in the ICU are compared, the pain scores in the absence and presence of an unavoidable painful stimulus should be tested in order to be able to study the sensitivity to change for each pain scale. In further studies, therefore, basal pain scores should be obtained together with intervention pain scores in order to evaluate and judge pain scales for different purposes (for example, at rest and during painful interventions).

Furthermore, the patients included in this study are characterized by a high percentage (73%) of post-cardiothoracic surgery patients and a 50% rate of mechanical ventilation, which was partly due to pain measurements before leaving the ICU, so extrapolation of the results to other ICUs may be limited. In addition, in our ICU, sedation levels are aimed at cooperative levels comparable to those of Kress and colleagues [4] (2000) and Brook and colleagues [27] (1999) whenever possible. Both of these characteristics of our ICU may have resulted in the relatively high percentage of responsive patients (66%) who were able to report pain using NRS and VAS. This should still be considered when the results of our study are extrapolated to other ICUs. On the other hand, in our study, there were no significant differences when the results were divided between 'cardiothoracic surgery' patients and 'non-cardiothoracic surgery' patients.

## Conclusion

The different scales show a high reliability, but observer-based evaluation often underestimates the pain, particularly in the case of high NRS values (≥4) rated by the patient. Therefore, whenever this is possible, ICU patients should rate their pain. In unresponsive patients, primarily the attending nurse involved in daily care should score the patient's pain. The BPS should be used only in conjunction with the NRS nurse to measure pain levels in the absence of painful stimuli.

## Key messages

• Especially unacceptably high patients' scores (Numerical Rating Scale [NRS] ≥4) are underestimated by nurses.

• Whenever possible, intensive care unit patients should rate their pain, which is the 'gold standard' in pain measurement.

• The Behavioral Pain Scale should be used only in conjunction with the NRS nurse to measure pain levels in the absence of painful stimuli.

## Abbreviations

BPS = Behavioral Pain Scale; ICU = intensive care unit; NRS = Numerical Rating Scale; r_s _= Spearman non-parametric rank correlation coefficient; RS = Ramsay Scale; VAS = Visual Analog Scale.

## Competing interests

The authors declare that they have no competing interests.

## Authors' contributions

SA carried out the practical work and drafted the manuscript. LG participated in the practical work and coordination, in revising the manuscript, and in the design and coordination of the study. AV participated in training the researchers for measuring pain. AB and DT participated in revising the manuscript critically. PB and HD participated in revising the manuscript critically and in the design and coordination of the study. SB carried out the statistical analysis. CK conceived of the study, participated in its design and coordination, and helped to draft the manuscript. All authors read and approved the final manuscript.
